# A 'Decrescendo' in a Woman With Ascending Paralysis: A Diagnostic Challenge

**DOI:** 10.7759/cureus.59479

**Published:** 2024-05-01

**Authors:** Ishwari Iyer, Rishav Sinha, Pradeep Kumar, Bryan Schaf, Leonard Berkowitz

**Affiliations:** 1 Internal Medicine, The Brooklyn Hospital Center, Brooklyn, USA; 2 Cardiology, The Brooklyn Hospital Center, Brooklyn, USA; 3 Pulmonary and Critical Care Medicine, The Brooklyn Hospital Center, Brooklyn, USA; 4 Infectious Diseases, The Brooklyn Hospital Center, Brooklyn, USA

**Keywords:** mitral insufficiency, severe mitral regurgitation, acute motor and sensory axonal neuropathy (amsan), blood culture negative endocarditis, blood culture negative infective endocarditis, guillain-barre syndrome (gbs)

## Abstract

Guillain-Barre Syndrome (GBS) is an autoimmune condition that causes muscular weakness and can be potentially life-threatening if not identified early. GBS is diagnosed definitively by cerebrospinal fluid (CSF) analysis and electromyographic (EMG) studies. Identifying illnesses that may have triggered GBS is crucial, as they could affect the course of the disease.

Our patient was a 27-year-old woman who developed lower extremity weakness a few days after being treated for a dental abscess. Laboratory and imaging studies ruled out central nervous system (CNS) lesions, myelopathies, and metabolic causes. Diagnosis was difficult due to inconclusive initial investigations, refusal of lumbar puncture, and delayed availability of EMG studies. Additionally, there were no identifiable triggers to support GBS as a diagnosis.

During the hospital course, the patient developed tachycardia with new electrocardiogram (EKG) changes. A transthoracic echocardiogram (TTE) showed suspicious vegetation, and a transesophageal echocardiogram (TEE) confirmed severe mitral regurgitation. The new valvular lesions and autonomic dysfunction with worsening lower extremity weakness increased our suspicion of GBS. Intravenous immunoglobulin (IVIG) was administered empirically, but she developed bulbar symptoms, prompting admission to the intensive care unit (ICU). A lumbar puncture performed at this time was negative for albumino-cytological dissociation and CNS infections. Signs of sepsis with valvular lesions raised concerns for infective endocarditis (IE). Due to recent treatment with antibiotics for dental abscess, a negative blood culture was a confounding factor in Duke's criteria, delaying the diagnosis of IE. Infectious disease experts suggested empirical treatment for suspected blood culture-negative infective endocarditis (BCNE) and valvular abscess. She was transferred to a cardiothoracic care facility for valvular surgery evaluation. EMG studies identified the patient's condition as the acute motor sensory axonal neuropathy (AMSAN) variant of GBS. The patient's antibodies tested positive for *Campylobacter jejuni (C. Jejuni) *immunoglobulin G (IgG). Since this indicates a past infection, it is uncertain whether *C. Jejuni *triggered the patient's GBS. However, new valvular vegetation and acute-onset lower extremity weakness make us hypothesize that BCNE may have triggered GBS.

## Introduction

Guillain-Barre Syndrome (GBS) is the leading cause of flaccid paralysis in developed countries following the eradication of polio [1]. GBS is an acute peripheral neuropathy that usually progresses rapidly and is linked to significant permanent impairment observed in 15-20% of cases [2]. Most cases of GBS in immunocompetent individuals are preceded by a respiratory or diarrheal illness. Antecedent infections caused by viruses and bacteria are common, and the most frequently identified pathogen is *Campylobacter jejuni (C. jejuni)*. However, it is important to note that only a minority of patients infected with *C. jejuni *will develop GBS [3]. In a previous report, a series of cases showed that infective endocarditis (IE) can be an unusual and rare trigger of GBS. Certain bacterial agents were identified through culture, but in some instances, IE was reported as blood culture-negative (BCNE) [4]. In situations where the microbe cannot be isolated, it often poses a diagnostic dilemma that could delay the initiation of specific antibiotics. This case describes an intriguing clinical scenario in which the diagnosis of GBS was challenging due to multiple confounding factors complicated by the parallel workup for new mitral regurgitation with sterile cultures.

## Case presentation

**Figure 1:**
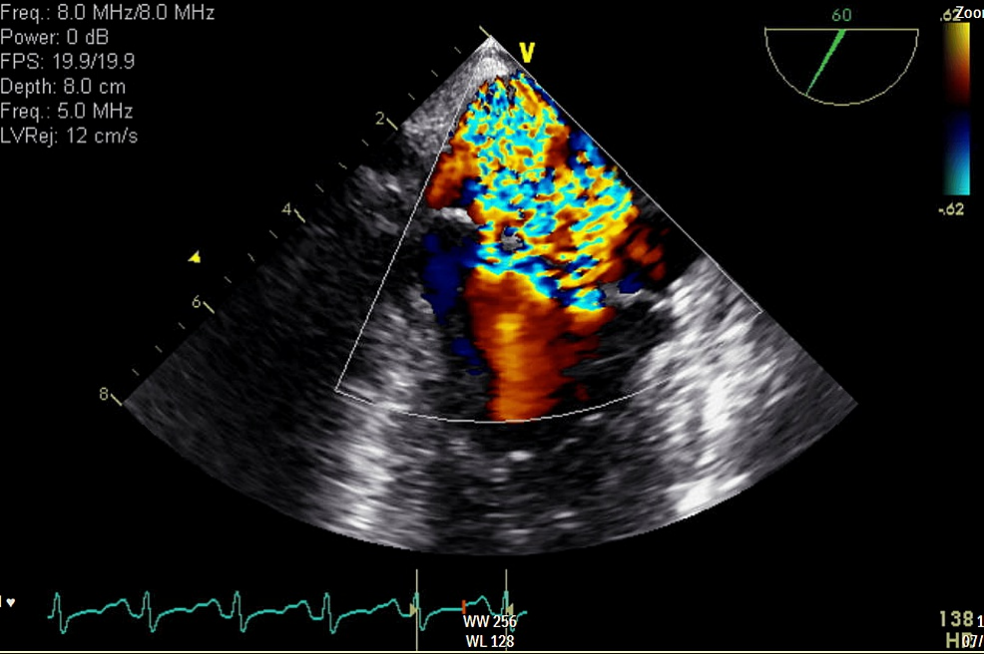
Transthoracic echocardiogram showing trace mitral regurgitation at the end of systole​

**Figure 2:**
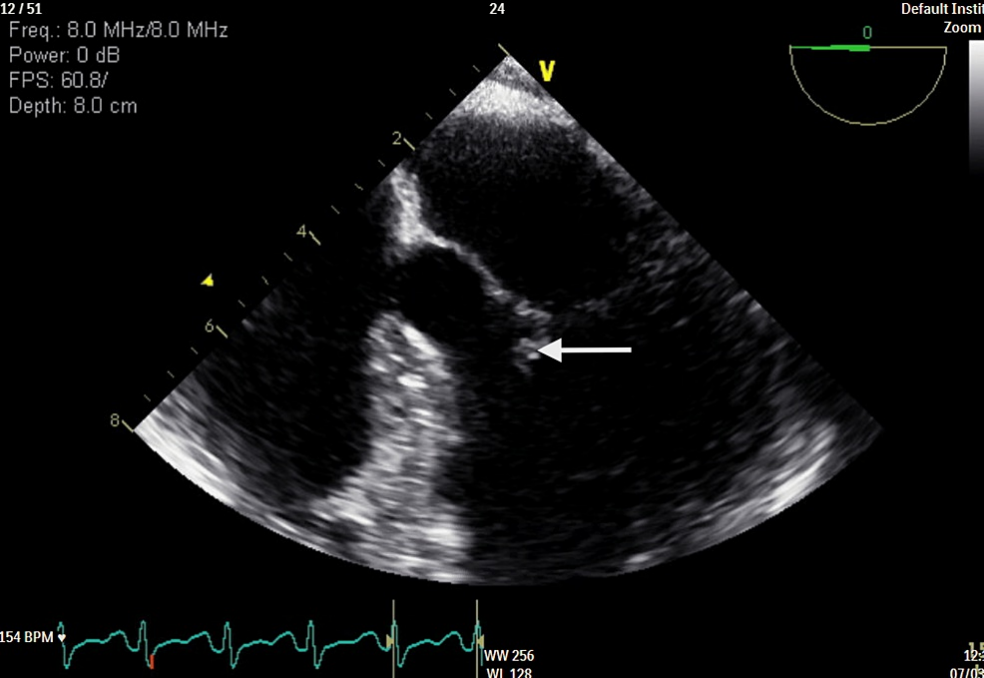
Transesophageal echocardiogram showing vegetation on the anterior leaflet of the mitral valve

**Figure 3:**
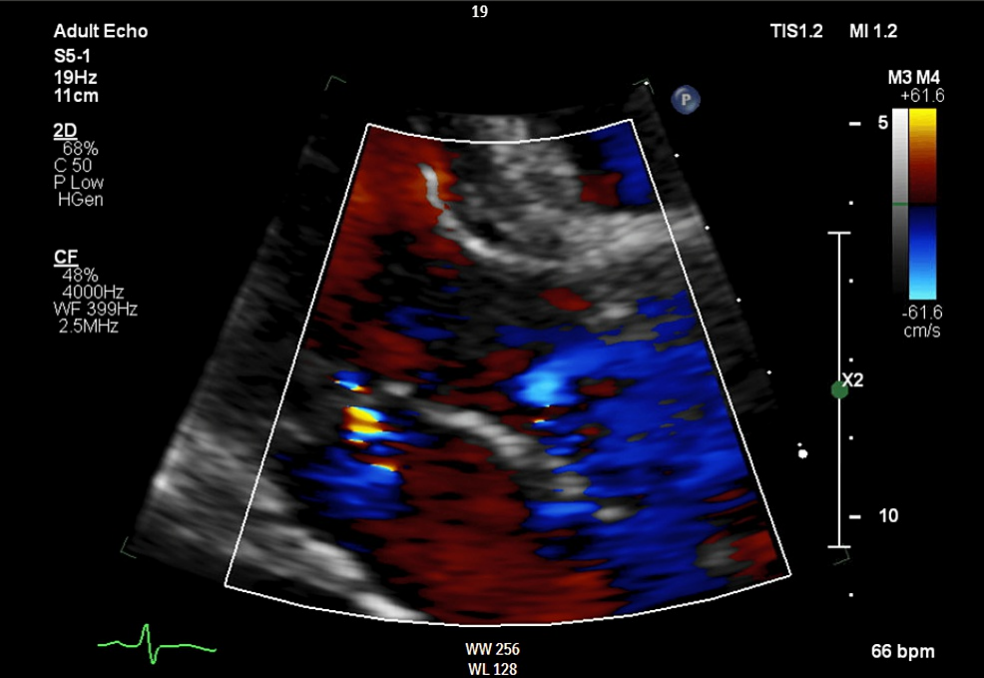
Transesophageal echocardiogram showing showing severe mitral regurgitation at the end of systole

A 27-year-old woman with a past medical history of anxiety disorder presented to the emergency department with progressive lower extremity weakness along with nausea and vomiting for a week. One month earlier, she had a dental abscess drained and was treated with a short course of antibiotics. She reported subjective fevers prior to hospitalization. She denied cigarette smoking, recreational drugs, and intravenous drug use. The patient was noted to be lethargic and oriented to person, place, and time. The bilateral lower extremities were hypotonic, and muscle strength was grade 2/5. Strength and reflexes in the upper extremities were normal. She had areflexia in bilateral knee and ankle joints. Sensations were intact. She did not exhibit facial asymmetry; extra-ocular movements were intact, and she had no dysphagia and a normal gag reflex. Romberg's test yielded negative results, and she did not exhibit dysdiadochokinesia. Examination of gait was limited by muscle weakness.  Labs were significant for hypokalemia (2.3 mmol/L) and leukocytosis. Antinuclear antibodies, human immunodeficiency virus, Cytomegalovirus, herpes simplex virus, Toxoplasma, porphyrins, and Lyme serologies were negative. Thyroid function tests were within normal limits. Non-contrast CT of the head and magnetic resonance imaging (MRI) of the brain and spine did not reveal any contrast enhancement, masses, or signs of compression. A lumbar puncture (LP) was not performed due to the patient’s refusal. Despite correcting electrolyte imbalances, her weakness continued to worsen, with no clear explanation of her symptoms. Her clinical course was complicated by episodic tachycardia and fluctuating blood pressure. Electrocardiogram (EKG) at this time showed sinus tachycardia with P-mitrale, which was not present on admission. Due to concerns about structural changes reflected on the EKG and recent dental abscess drainage, a transthoracic echocardiogram (TTE) was performed (Figure 1), followed by a transesophageal echocardiogram (TEE) (Figure 2, Figure 3). There were no past records of cardiac conditions for this patient. She reported no prior history of shortness of breath, chest pain, dizziness, fainting, or sudden cardiac death in her family.

TTE revealed mitral valve lesions. Signs of sepsis and valvular lesions on TTE raised suspicion of infective endocarditis, leading to further evaluation with TEE. TEE confirmed mitral valve vegetations with severe mitral regurgitation. New valvular lesions with autonomic dysfunction in the setting of worsening lower extremity weakness increased our suspicion of GBS, leading to a decision to administer intravenous immunoglobulin (IVIG) empirically. Despite receiving a complete course of IVIG, she developed bulbar symptoms warranting close observation in the intensive care unit (ICU). LP performed at this time showed protein levels of 25 mg/dL and 5 mg/dL of leukocytes. Spinal fluid culture and viral encephalitis panel were negative. Signs of sepsis in the presence of valvular lesions raised concerns for infective endocarditis and valvular abscess. According to the modified Duke's criteria, she met two major criteria for IE, with echocardiogram evidence of mitral valve vegetations and significant new valvular regurgitation compared to previous imaging. Due to resource constraints, serological tests were not performed. Ceftriaxone was administered for suspected culture-negative endocarditis for a total of four weeks in accordance with Infectious Disease expertise.

She was then transferred to a facility with advanced cardiac care to be evaluated for mitral valve repair. A repeat MRI of the lumbar spine revealed a new enhancement in the cauda equina. An electromyogram (EMG) demonstrated severe generalized axonal sensorimotor polyneuropathy consistent with acute motor and sensory axonal neuropathy (AMSAN), a variant of GBS. She was considered ineligible for mitral valve surgery due to her neurological condition. After gradual improvement in her strength and recovery from bulbar deficits, she was discharged to acute rehabilitation.

## Discussion

In this case, the diagnoses of both GBS and IE were complex due to numerous confounding factors, including recent antibiotics use, which played a role in their presentations. Considering the temporality of the development of paralysis and valvular dysfunction with suspicious vegetations in the heart, we hypothesize that these two conditions may be related. The differential diagnosis to the patient’s initial presentation included muscle weakness due to hypokalemia, myelopathy, infections such as meningitis or epidural abscess, an acute flare-up of autoimmune myopathies, stroke, and structural lesions such as tumors. Symptoms persisted despite the repletion of electrolytes. The auto-immune workup was negative. Lack of upper motor neuron signs ruled out myelopathy, and the meningitis panel was negative on an LP performed later in the course. A negative MRI of the brain and spinal cord ruled out a tumor, stroke, and epidural abscess. Initial refusal of LP by the patient, and the delayed availability of EMG studies impeded the diagnostic process. Additionally, there were no identifiable triggers for suspected GBS.

LP that was performed two weeks after the onset of symptoms was negative for albumino-cytological dissociation. Given the high sensitivity of LP for GBS at this period, negative results served as a setback in our diagnosis [5]. It is, however, vital to note that completing a five-day course of IVIG before LP may have interfered with the diagnostic yield. Ascending paralysis, areflexia, subsequent cranial nerve involvement, and autonomic dysfunction increased the clinical suspicion of GBS based on the National Institute of Neurological Disorders and Stroke (NINDS) criteria. This led to the persuasion of GBS as a working diagnosis despite negative tests. Additionally, Brighton criteria assisted in the process due to the heterogeneity of its presentation. There was a diagnostic certainty of level 2, which has shown to have a 96% sensitivity for GBS in certain studies [6]. There was a delay in obtaining the results for *C. Jejuni *serology, making it a challenge to identify the likely trigger of this patient’s symptoms. However, the results eventually showed the presence of immunoglobulin-G antibodies, indicative of a past infection; thus making *C. jejuni *less likely to be a trigger. A decision was made to obtain a transthoracic echocardiogram due to new EKG changes reflecting left atrial enlargement in view of a recent dental abscess and signs of sepsis. The presence of mitral lesions suspicious for vegetation and mitral insufficiency leads to a parallel workup for IE. It is important to note that the recent administration of antibiotics would have interfered with the result of blood cultures, adding to the intricacy in the diagnosis of this case. Interestingly, *Streptococcus viridans* isolated in this patient's recent dental abscess is seen to be associated with blood culture-negative endocarditis (BCNE) [7]. IE with negative cultures may either be due to recent antibiotic exposure, fastidious microorganisms, or microorganisms that grow intracellularly and cannot be cultured. Serological tests for bacteria play a crucial role in diagnosing BCNE [8]; however, they can result in false positive results, as microorganism-specific immunoglobulin-M tends to be present in the serum for an extended period [9].

Obtaining serological testing, along with an initial set of blood cultures, could aid in the initiation of specific antibiotics and in reducing the duration of hospitalization. Histopathology, often obtained during valvular surgery, can provide a definitive diagnosis. Duke’s criteria may be a poor predictor of IE in BCNE [10]. For IE caused by *Coxiella*, the modified Duke’s criteria not only include a new valvular regurgitation and signs of IE on echocardiogram but also a positive serology as an alternative to blood cultures. Additionally, a positive serology qualifies as a minor criterion in diagnosing BCNE [8]. The treatment of BCNE depends on the likely microorganism that caused it. In the absence of a suspicious microorganism, BCNE may be treated with vancomycin and cefepime empirically. BCNE, which is suspected to have a subacute onset, is treated with penicillin and gentamicin. Vancomycin is added to high-risk groups such as patients with a history of intravenous drug use. Recent prosthetic valve insertion warrants vancomycin along with gentamicin with consideration of rifampin [11]. In our patient, besides IE, there were concerns of mitral valve abscess due to the history of recent dental incision and drainage. With infectious disease expertise, ceftriaxone monotherapy was the drug of choice based on microorganisms isolated from her dental abscess. This includes *S. viridans,* which is one of the agents implicated in BCNE.

An MRI performed a few weeks after symptoms onset showed enhancement of the cauda equina, which is a classical sign of GBS [12]. EMG confirmed and characterized the condition as the AMSAN variant of GBS. GBS is classified into acute motor axonal neuropathy (AMAN) and acute motor and sensory axonal neuropathy (AMSAN) based on the location of injury by molecular mimicry on neurons. These subtypes of GBS differ in the progression, recovery, and prognosis [13].

Various measures can be taken to hinder the advancement of GBS in similar cases. Utilizing clinical resources, such as the Brighton criteria, helps improve diagnostic accuracy [14]. Early confirmation with EMG assists in identifying the subtype of GBS, which aids in both treatment and prognostication. Prompt recognition of IE and its association with GBS is crucial. Serological tests should be performed with blood cultures if there is a high clinical suspicion for IE, as they help identify atypical organisms. When there is a strong suspicion of IE, and blood cultures yield negative results, it is important to hold a multidisciplinary discussion regarding the necessity of performing a TEE. Furthermore, a collaborative, interdisciplinary approach is important in understanding the case and its complications from the perspective of different specialties.

## Conclusions

The prolonged diagnostic uncertainty due to atypical presentations of both IE and GBS makes this case unique. This case emphasizes the importance of utilizing standardized diagnostic criteria in conjunction with a clinical assessment of patient symptoms and history to aid in the diagnosis of medical conditions that present atypically. Effective interdisciplinary collaboration is an essential component to ensure prompt escalation of care, which has been highlighted in this case.
